# Activation of the NLRP3 inflammasome in lipopolysaccharide-induced mouse fatigue and its relevance to chronic fatigue syndrome

**DOI:** 10.1186/s12974-016-0539-1

**Published:** 2016-04-05

**Authors:** Zi-Teng Zhang, Xiu-Ming Du, Xiu-Juan Ma, Ying Zong, Ji-Kuai Chen, Chen-Lin Yu, Yan-Gang Liu, Yong-Chun Chen, Li-Jun Zhao, Guo-Cai Lu

**Affiliations:** Department of Health Toxicology, College of Tropical Medicine and Public Health, Second Military Medical University, Shanghai, 200433 China; Laboratory Animal Center, Second Military Medical University, Shanghai, 200433 China; Department of Respiratory Medicine, Changhai Hospital, Second Military Medical University, Shanghai, 200433 China

**Keywords:** NLRP3 inflammasome, LPS, Chronic fatigue syndrome, NLRP3 knockout mice, IL-1β

## Abstract

**Background:**

The NLRP3 inflammasome (NOD-like receptor family, pyrin domain containing 3) is an intracellular protein complex that plays an important role in innate immune sensing. Its activation leads to the maturation of caspase-1 and regulates the cleavage of interleukin (IL)-1β and IL-18. Various studies have shown that activation of the immune system plays a pivotal role in the development of fatigue. However, the mechanisms underlying the association between immune activation and fatigue remained elusive, and few reports have described the involvement of NLRP3 inflammasome activation in fatigue.

**Methods:**

We established a mouse fatigue model with lipopolysaccharide (LPS, 3 mg/kg) challenge combined with swim stress. Both behavioural and biochemical parameters were measured to illustrate the characteristics of this model. We also assessed NLRP3 inflammasome activation in the mouse diencephalon, which is the brain region that has been suggested to be responsible for fatigue sensation. To further identify the role of NLRP3 inflammasome activation in the pathogenesis of chronic fatigue syndrome (CFS), NLRP3 KO mice were also subjected to LPS treatment and swim stress, and the same parameters were evaluated.

**Results:**

Mice challenged with LPS and subjected to the swim stress test showed decreased locomotor activity, decreased fall-off time in a rota-rod test and increased serum levels of IL-1β and IL-6 compared with untreated mice. Serum levels of lactic acid and malondialdehyde (MDA) were not significantly altered in the treated mice. We demonstrated increased NLRP3 expression, IL-1β production and caspase-1 activation in the diencephalons of the treated mice. In NLRP3 KO mice, we found remarkably increased locomotor activity with longer fall-off times and decreased serum IL-1β levels compared with those of wild-type (WT) mice after LPS challenge and the swim stress test. IL-1β levels in the diencephalon were also significantly decreased in the NLRP3 KO mice. By contrast, IL-6 levels were not significantly altered.

**Conclusions:**

These findings suggest that LPS-induced fatigue is an IL-1β-dependent process and that the NLRP3/caspase-1 pathway is involved in the mechanisms of LPS-induced fatigue behaviours. NLRP3/caspase-1 inhibition may be a promising therapy for fatigue treatment.

**Electronic supplementary material:**

The online version of this article (doi:10.1186/s12974-016-0539-1) contains supplementary material, which is available to authorized users.

## Background

Fatigue makes a significant contribution to the global disease burden. A spectrum of fatigue-related syndromes, known as chronic fatigue syndrome (CFS), includes cognitive problems, sleep disturbances, malaise and gastrointestinal symptoms [1]. CFS is characterized by persistent and severe fatigue [2]. In addition to fatigue, CFS patients also complain of somatic symptoms, such as irritable bowel syndrome (IBS) and migraines, and these symptoms can be worsened by stress and over exercise [3–5]. According to reports published by the US Centres for Disease Control and Prevention (CDC), the incidence of CFS may be as high as 1 % in the US population, and the female to male ratio is 4:1 [6].

Although many studies regarding fatigue have been published in recent decades, the exact pathophysiological mechanisms of fatigue are not well delineated. Several lines of evidence indicate that non-viral pathogen entry into the body induces the expression of cytokines in the central nervous system (CNS) [7]. Recent investigations have revealed that the interaction between inflammatory pathways and the neuroendocrine system is associated with the manifestation of symptoms such as pain, fatigue, impaired memory and depression, which largely characterize at least some patients suffering from CFS [8]. Induction of interleukin (IL)-1β expression and elevated inflammatory mediators in the brain are prerequisites for decreased locomotor activity and other fatigue-related behaviours [9, 10]. Moreover, many CFS patients demonstrate abnormal hypothalamic-pituitary-adrenal (HPA) axis activity [11, 12]. In patients with fatigue-predominant CFS, there was significant enhancement in nocturnal ACTH-adrenal signalling and marginally increased inhibitory feedback compared with that of healthy controls [13]. In addition, fatigue improvement (defined as a reduction in fatigue score) has been reported in response to low-dose hydrocortisone therapy [14]. The involvement of HPA axis dysfunction suggested that the diencephalon was the brain region responsible for fatigue sensation, although the molecular/cellular mechanisms of this process were still unclear.

In recent years, increased attention has been paid to the importance of the cytosolic signalling pathways of inflammation [15]. Among these, the NOD-like receptor pyrin domain containing 3 (NLRP3) inflammasome is of particular interest. The NLRP3 inflammasome can be activated by a number of different stimuli known as danger-associated molecular patterns (DAMPs) [16]. Among these stimuli, the production of reactive oxygen species (ROS) and mitochondrial dysfunction are also major contributors to the development of fatigue-related sensations [17]. Once activated, the NLRP3 inflammasome can trigger increased induction of caspase-1 cleavage and IL-1β production [18]. Cordero et al.’s study reveals that coenzyme Q10 (CoQ10) deficiency can induce NLRP3 inflammasome activation in the peripheral blood mononuclear cells (PBMCs) of CFS patients [19].

However, whether the NLRP3 inflammasome in the CNS is involved in the development of fatigue sensation remains elusive. Therefore, the present study was designed to assess NLRP3 inflammasome activation status and reveal its potential involvement in the pathogenesis of peripheral immune system-induced fatigue. Behavioural and biochemical alterations were also measured to evaluate fatigue in a mouse model of CFS.

## Methods

### Chemicals and reagents

Lipopolysaccharide (LPS) from *Escherichia coli* 0111:B4 (catalogue: L2630-10MG) was purchased from Sigma-Aldrich (St. Louis, MO, USA); anti-mouse NLRP3 Ab (AG-20B-0014) was from AdipoGen Corp. (San Diego, CA); anti-mouse caspase-1 p10 Ab and anti-mouse actin Ab were from Santa Cruz Biotechnology, Inc. (Dallas, Texas); and anti-mouse IL-1β Ab was from Cell Signaling Technology (Beverly, MA). For immunofluorescence staining, anti-mouse caspase-1 p10 Ab (sc-22166) was from Santa Cruz Biotechnology, Inc. (Dallas, Texas); anti-mouse IL-1β Ab (ab9722) was from Abcom (Massachusetts, USA); Alexa Fluor 488 Goat anti-Rabbit, Alexa Fluor 555 Goat anti-Mouse and Alexa Fluor 647 Goat anti-Rabbit were from Life Technology (Shanghai, People’s Republic of China). The reagents listed above were prepared and used according to the manufacturer’s instructions.

### Animals

C57BL/6 female mice (wild-type (WT) control) weighing 20–23 g were purchased from Shanghai Super-B&K Laboratory Animal Corp. Ltd. C57BL/6 NLRP3 KO female mice were provided by the Model Animal Research Centre of Nanjing University (AAALAC accredited). The animals were housed in a pathogen-free animal facility with 12-h light and 12-h dark cycles (8:00–20:00 light and 20:00–8:00 dark) with free access to food and water.

### Treatment conditions

To induce the CFS model, the mice were injected intraperitoneally with 3 mg/kg of LPS or normal saline [20]. Twenty-four hours after injection, the mice were subjected to a swim stress test for 20 min. The mice were forced to swim individually in a transparent plastic square jar (25 cm × 31 cm) containing 15-cm-deep water at 23 ± 2 °C. A light lead sinker (5 % of the body weight) was attached to the tail root of each mouse. The mice were then removed from the pool and dried with a clean towel. The pool water was replaced after each session. The following four groups of mice were created to test the effect of LPS treatment during the swim test: (a) control (saline)/no swim; (b) LPS/no swim; (c) control/swim; and (d) LPS/swim. To confirm our findings and test the importance of NLRP3 inflammasome activation in the CFS model, the weight-matched NLRP3 KO female mice were also subjected to LPS challenge and swim stress testing. Normal saline was administered to the control mice. Each group contained 8–10 animals. All of the animal studies were approved by the Ethics Committee of the Second Military Medical University, and all procedures were performed in compliance with the Guideline for Care and Use of Laboratory Animals published by the National Institutes of Health, USA.

### Sample collection

The mice were anesthetized with pentobarbital sodium (100 mg/kg) and then sacrificed. The whole brain was rapidly extracted from the animals and placed on ice. The diencephalon tissue (mainly the hypothalamus) was quickly dissected and then frozen in liquid nitrogen. A representative image of the dissected region is shown in Additional file 1: Figure S1. Blood was collected by cardiac puncture and centrifuged at 3000*g* for 15 min at 4 °C (Eppendorf 5801R centrifuge, Germany), and the serum was collected to measure the levels of certain biochemical mediators in the serum. The right quadriceps femoris was immediately dissected and weighed. Muscle samples were manually homogenized with a glass homogenizer using ice-cold physiological saline. Homogenates were centrifuged for 15 min at 3000*g* (Eppendorf 5801R centrifuge, Germany), and the supernatants were collected to determine malondialdehyde (MDA) concentrations.

### Assessment of behavioural parameters

The behavioural parameters consisted of locomotor activity assessment and a rota-rod test. After the experimental procedure, the animals were dried with a clean towel and allowed to rest in their original cages for 30 min. The locomotor activity was monitored for a total of 1 h using the Neuroscience Behaviour Mouse Cage Rack System (ShangHai Biowill Co., Ltd, Shanghai, China). This system contained 12 individual automated cages, which each continuously recorded the animal’s movement using a micro video camera. The video was then analysed using the native Motor Monitor software (ShangHai Biowill Co., Ltd). The data were collected and analysed in 10-min time intervals. For the rota-rod test, the animals were first conditioned at a constant speed of 10 rpm for a period of 5 min. The animals that failed the first conditioning were given two additional conditioning periods. For the assessment, the mice were individually placed on the rota-rod, which was adjusted to a speed of 30 rpm. The fall-off time was recorded for each mouse, and the longest period any animal could be maintained on the rod was 600 s.

### Assessment of biochemical mediators

Mouse serum levels of lactic acid and MDA were determined using commercial assay kits (Xi Tang Biotechnologies Co., Ltd, Shanghai, China). Muscle levels of MDA were measured as well. IL-1β and IL-6 mouse serum levels were measured with ELISA kits (Dakewe Biotech Company Ltd., Shenzhen, China) according to the manufacturer’s instruction.

### Real-time PCR analysis for NLRP3 and pro-IL-1β mRNA

The total RNA was extracted from frozen diencephalon tissues using Trizol reagent (Life Technologies, USA). The homogenate was mixed with 200 μl chloroform and then centrifuged at 12,000*g* for 15 min at 4 °C. The aqueous phase (approximately 0.5 ml of the upper layer) was precipitated with an equal volume of isopropanol and centrifuged at 12,000*g* for 10 min at 4 °C. The final total RNA pellet was resuspended in 20 μl DEPC water. Reverse transcription was performed with 1 μg total RNA using the Transcriptor First Strand cDNA Synthesis Kit (Roche Ltd, Swiss). A total of 2 μl first-strand cDNA solution was used for real-time RT-PCR in combination with a Fast Start Universal Probe Master Mix (ROX). All experiments were run in triplicate. The real-time PCR was run on an Applied Biosystems 7500 Real-Time PCR System (Life Technologies Corporation, USA). The primers used for RT-PCR are listed in Table 1. The threshold cycle (CT) of the target product was normalized to that of the internal standard GADPH.

**Table 1:** Primer used for real-time PCR in this study

Genes	Forward primer(5'→3')	Reverse primer(5'→3')
NLRP3	ACCAGCCAGAGTGGAATGAC	ATGGAGATGCGGGAGAGATA
Pro-IL-1β	CTCACAAGCAGAGCACAAGC	TCCAGCCCATACTTTAGGAAGA
GADPH	GTGTTTCCTCGTCCCGTAGA	AATCTCCACTTTGCCACTGC

### Western blot analysis

The brain samples were lysed with RIPA Lysis Buffer (Aidlab Biotechnologies Co., Ltd, Beijing, China) supplemented with a protease inhibitor “cocktail”, and protein concentrations in the extracts were measured using the BCA Protein Assay Kit (Aidlab Biotechnologies Co., Ltd, Beijing, China). An equal amount of each extract was separated by SDS-PAGE and then transferred onto nitrocellulose membranes. The membranes were blocked in a 5 % non-fat milk solution for 2 h at 24 °C. The blots were then incubated with primary antibodies for 24 h at 4 °C and then incubated with secondary antibodies for 2 h at 4 °C. Full film scans of the Western blot data were obtained with an Amersham Imager 600 (GE Healthcare Bio-Sciences AB, Sweden). The protein expression levels were quantified by measuring band intensities using ImageJ software (NIH, USA). The band intensity values of the proteins of interest were normalized to that of actin.

### Immunofluorescence staining

After anesthetisation, the mice were transcardially perfused with normal saline (0.9 %), and brain tissues were fixed in a fresh 4 % paraformaldehyde solution (pH 7.4) at 4 °C. Coronal sections (30 μm) containing the diencephalon were prepared for immunofluorescence staining. Primary antibodies against NLRP3, caspase-1 and IL-1β were also used to delineate respective inflammasome components. DAPI (4′,6-diamidino-2-phenylindole) was used for nuclear staining. Alexa Fluor 488, Alexa Fluor 555 and Alexa Fluor 647 were used as secondary antibodies. Images of the stained specimens (five mice per group) were captured using an Olympus Research Inverted System Microscope IX71 (Olympus, Japan).

### Statistical analysis

The results were measured and expressed as the mean ± SEM. Locomotor activity data were first analysed by a repeated measures analysis of variance (ANOVA) to determine the effects of the treatment. Other variations among groups were analysed by a univariate ANOVA followed by Dunnett’s test to compare any two groups. The comparisons made between two groups were evaluated using Student’s independent *t* test. All statistical analyses were performed using SPSS 21 software. Statistical significance was defined as *p* < 0.05.

## Results

### Effects of LPS treatment and swim stress testing on behavioural and biochemical parameters in mice

**Fig. 1:**
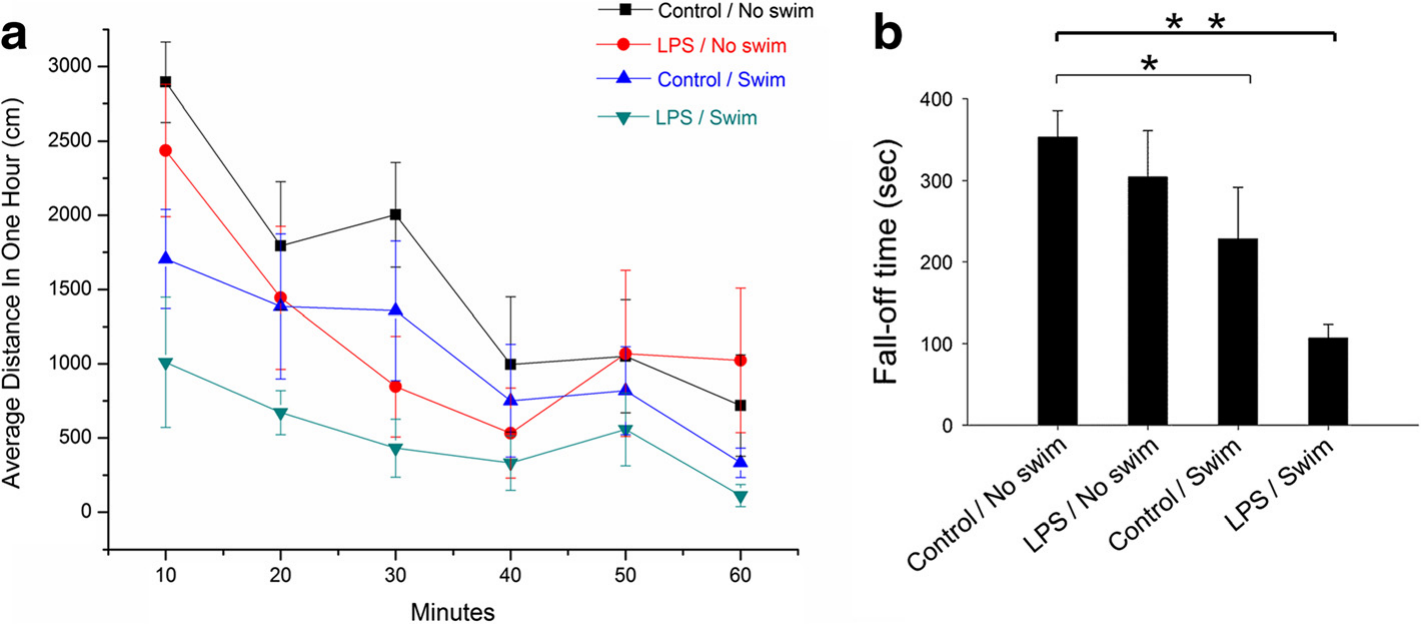
Behavioural alterations in LPS/swim stress-treated mice. **a** LPS/swim stress treatment resulted in decreased locomotor activity. Locomotor activity was monitored at 10-min intervals for 1 h. **b** Reduced fall-off time in LPS/swim stress and control/swim stress groups during the rota-rod test. **p < 0.01, *p < 0.05 compared to control/no swim stress mice (ANOVA). (*n* = 8–10 mice per group)

Behavioural alterations were assessed by locomotor activity and rota-rod testing [21]. As shown in Fig. 1a, the locomotor activity of each mouse was monitored for a 1-h observation period, and the data were collected within 10-min intervals. When compared to the control saline/no swim group, the LPS/swim-treated mice showed significantly reduced locomotor activity (*p* < 0.01), whereas no difference was found in either the LPS/no swim group (*p* = 0.89) or the control/swim group (*p* = 0.23). There was also a significant difference in locomotor activity between the LPS/swim group and the LPS/no swim group (*p* = 0.043).

In the rota-rod test, our results indicated that both the control/swim and LPS/swim groups (228.43 ± 63.17 s, *p* < 0.05; 106.71 ± 17.14 s, *p* < 0.01; respectively in Fig. 1b) displayed significantly decreased fall-off time when compared with that of the control/no swim mice (353.43 ± 52.53 s). There was also a significant difference between the fall-off time of the LPS/swim group and that of the LPS/no swim group (*p* < 0.05). Consistent with the locomotor activity results, LPS treatment alone did not affect the fall-off time (*p* = 0.35).

**Fig. 2:**
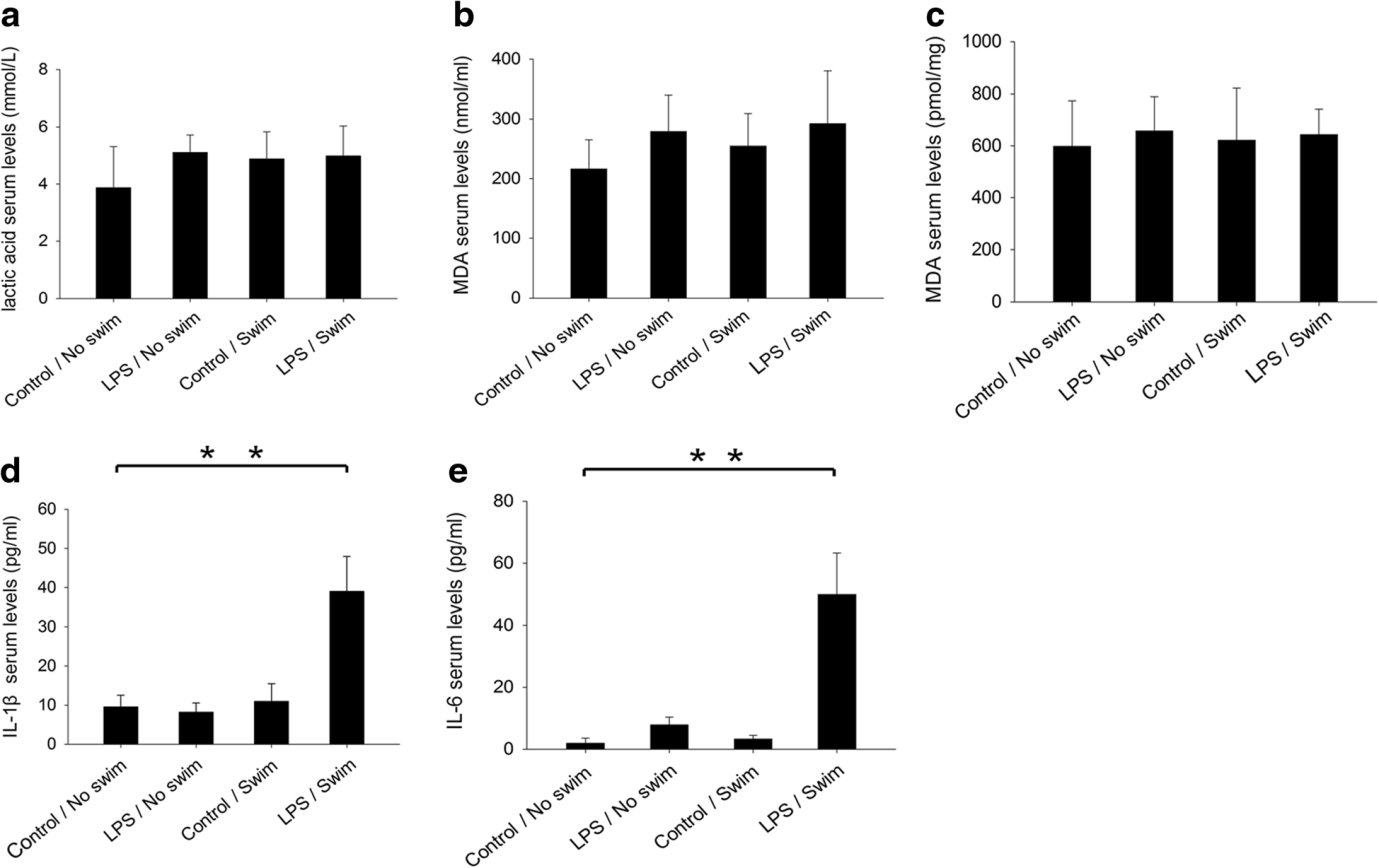
Biochemical alterations in LPS/swim stress-treated mice. Mice were sacrificed 30 min after the forced swim, and biochemical parameters were measured. There was no significant change in lactic acid (**a**) or MDA levels (**b** serum; **c** muscle). LPS/swim stress challenge significantly increased IL-1β (**d**) and IL-6 (**e**) levels. The results are expressed as the mean ± SEM. **p < 0.01 compared to control/no swim mice (ANOVA). (*n* = 8–10 mice per group)

Regarding serum biochemical parameters, we compared the lactic acid serum levels among the four groups and found no significant difference (*p* = 0.30, Fig. 2a). Assessment of MDA levels showed that although LPS/swim treatment enhanced the production of MDA when compared with that of the control/no swim group (292.31 ± 88.23 nmol/ml vs. 216.50 ± 48.78 nmol/ml, Fig. 2b), the difference was not significant (*p* = 0.16). Similarly, LPS/swim treatment increased muscle MDA levels, but we detected no significant difference in comparison with the muscle tissue MDA level in the control/no swim mice (0.62 ± 0.08 pmol/mg vs. 0.60 ± 0.06 pmol/mg, Fig. 2c).

LPS challenge combined with swim stress significantly increased the serum IL-1β concentration (39.00 ± 8.93 pg/ml) when compared with that of the control/no swim-treated mice (8.18 ± 2.36 pg/ml, *p* < 0.01, Fig. 2d). LPS challenge or swim stress alone did not affect serum IL-1β levels. Similar results were also found for serum IL-6 levels. The LPS/swim mice produced remarkably more serum IL-6 (49.93 ± 13.41 pg/ml) than did the control/no swim mice (1.95 ± 1.66 pg/ml, *p* < 0.01, Fig. 2e). LPS challenge or swim stress alone did not affect serum IL-6 levels.

### Effects of LPS treatment and swim stress on NLRP3 inflammasome activation in the mouse diencephalon

**Fig. 3:**
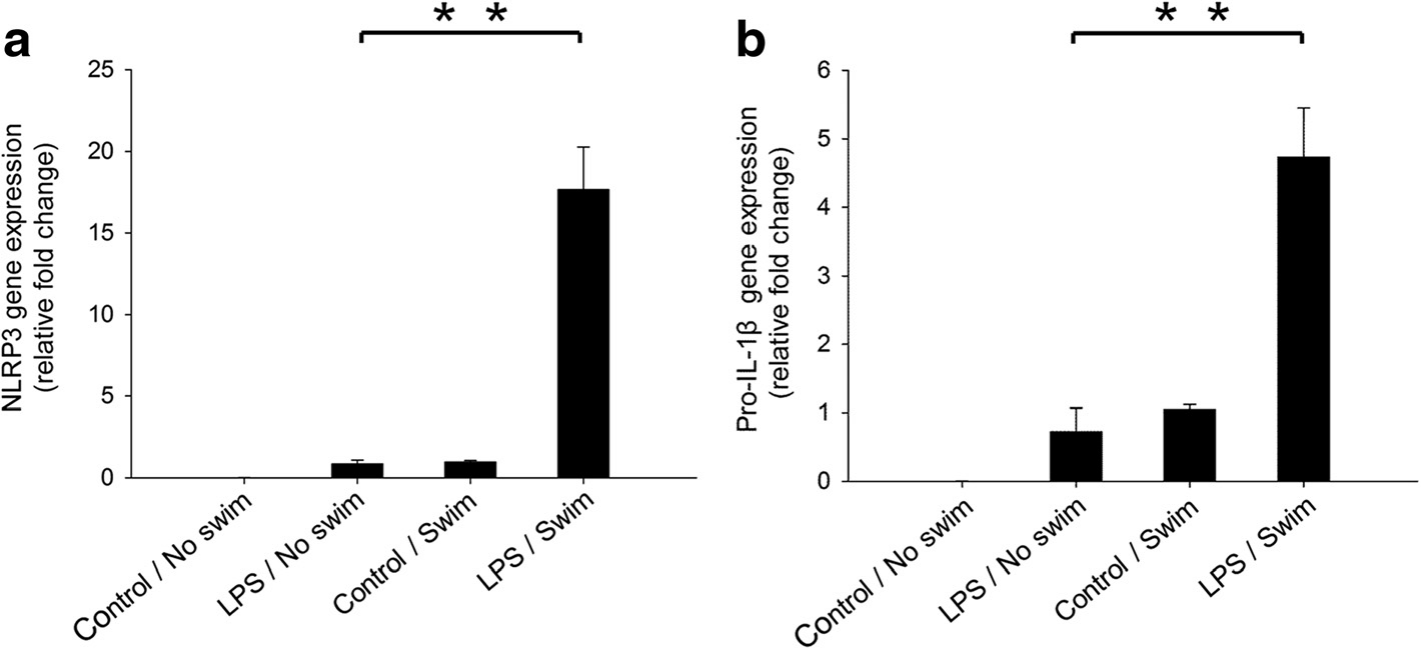
NLRP3 and pro-IL-1β mRNA levels were significantly elevated in the mouse diencephalon after LPS/swim treatment. NLRP3 mRNA levels (**a**) and pro-IL-1β mRNA levels (**b**) were calculated using the ΔΔC_t_ method, ΔΔC_t_ = target group (CT of target genes − CT of GADPH) − control group (CT of target genes − CT of GADPH), and data were expressed as fold change (*n* = 4–6). **p < 0.01

**Fig. 4:**
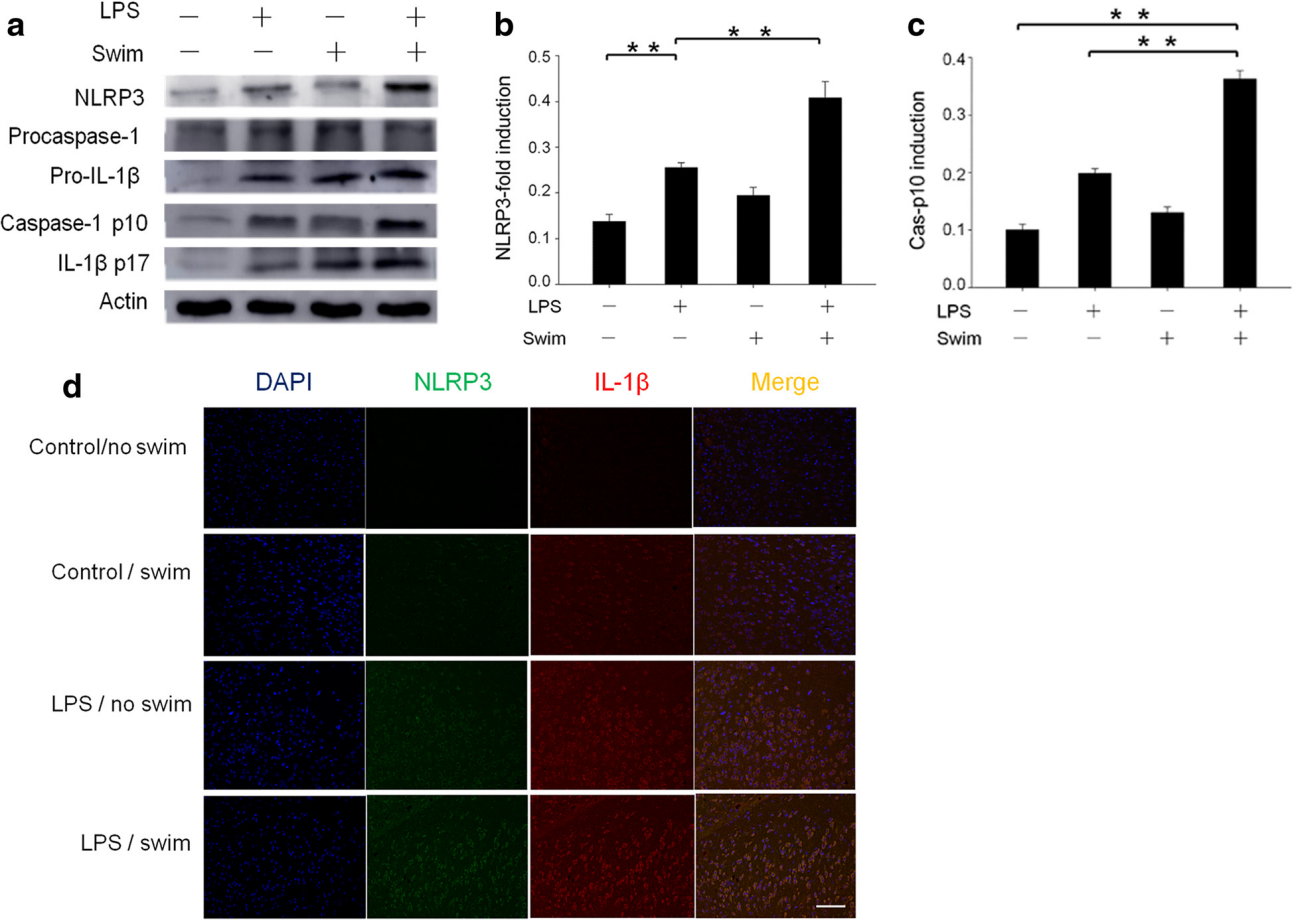
The NLRP3 inflammasome was activated in the mouse diencephalon following LPS/swim challenge. The samples came from one mouse per experiment, and the results are representative of three independent experiments (**a**). NLRP3 expression levels (**b**) and Cas-p10 induction (**c**) were quantified by measuring band intensities using ImageJ software. The values were normalized to β-actin. **d** DAPI (*blue*), NLRP3 (*green*) and IL-1β (*red*) immunofluorescent staining in diencephalon sections; *scale bar*, 20 μm. The merged image showed the co-localisation of the NLRP3 and IL-1β proteins. **p < 0.01. Data are represented as the mean ± SEM of three samples

NLRP3 protein expression is crucial for the formation and activation of the NLRP3 inflammasome [22]. Therefore, we investigated the effect of LPS treatment and swim stress on NLRP3 inflammasome expression. In the control/no swim mice, there was almost no detectable mRNA expression of NLRP3 or pro-IL-1β (Fig. 3). However, LPS administration significantly elevated NLRP3 and pro-IL-1β mRNA expression in the brains of LPS-treated mice. The LPS/swim mice exhibited significantly increased NLRP3 and pro-IL-1β mRNA levels in the diencephalon when compared with that of the LPS/no swim mice (both *p* < 0.01, Fig. 3). Western blot analysis demonstrated that LPS treatment alone greatly increased NLRP3 production. Our results indicated that swim stress could significantly augment this effect. Compared with the LPS/no swim group, the LPS/swim group showed considerably increased NLRP3 expression (*p* < 0.01, Fig. 4b). Consistent with the NLRP3 expression data, increased amounts of cleaved caspase-1 were also detected in the LPS/swim-treated mice when compared with the LPS/no swim mice (*p* < 0.01, Fig. 4c), suggesting that the NLRP3 inflammasome was intensely activated in the diencephalon region. By contrast, swim stress alone was unable to cause such an effect. There was no significant difference in NLRP3 or cleaved caspase-1 expression levels between the control/no swim group and the control/swim group (*p* = 0.13 for NLRP3 expression and *p* = 0.11 for cleaved caspase-1 expression). Similar results were obtained using immunofluorescence analysis for increased NLRP3 and IL-1β staining in the diencephalon tissues from LPS/swim mice. Co-localized NLRP3 and IL-1β expression was also significantly increased in the LPS/swim mice compared with any other groups (Fig. 4d).

### Effects of NLRP3 deficiency on behavioural and biochemical parameters in LPS/swim stress-treated mice

**Fig. 5:**
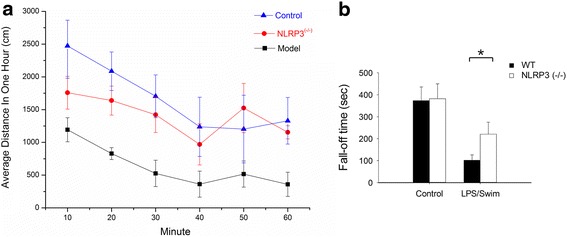
Effect of NLRP3 deficiency on behavioural parameters in the CFS model. **a** Increased locomotor activity in NLRP3 KO mice compared with wild-type mice. **b** Increased fall-off time in NLRP3 KO mice following LPS/swim challenge compared with wild-type mice in the rota-rod test. *p < 0.05 (independent *t* test). (*n* = 8–10 mice per group)

To determine the role of the NLRP3 inflammasome and its activation during the pathogenesis of LPS-induced CFS, the weight-matched NLRP3 KO mice were also subjected to LPS/swim challenge and the same behavioural and biochemical parameters were observed. Locomotor activity levels between the control mice and CFS model mice were significantly different, which confirmed the reliability of the LPS/swim treatment CFS model (*p* < 0.01). Following LPS/swim challenge, the NLRP3 KO mice exhibited significantly improved motor performance when compared with wild-type mice (*p* < 0.05, Fig. 5a). In the rota-rod test, the NLRP3 KO mice showed a slightly longer fall-off time than did the wild-type mice (*p* < 0.05, Fig. 5b), although there was still a significant difference when compared with that of non-LPS/no swim-treated mice.

**Fig. 6:**
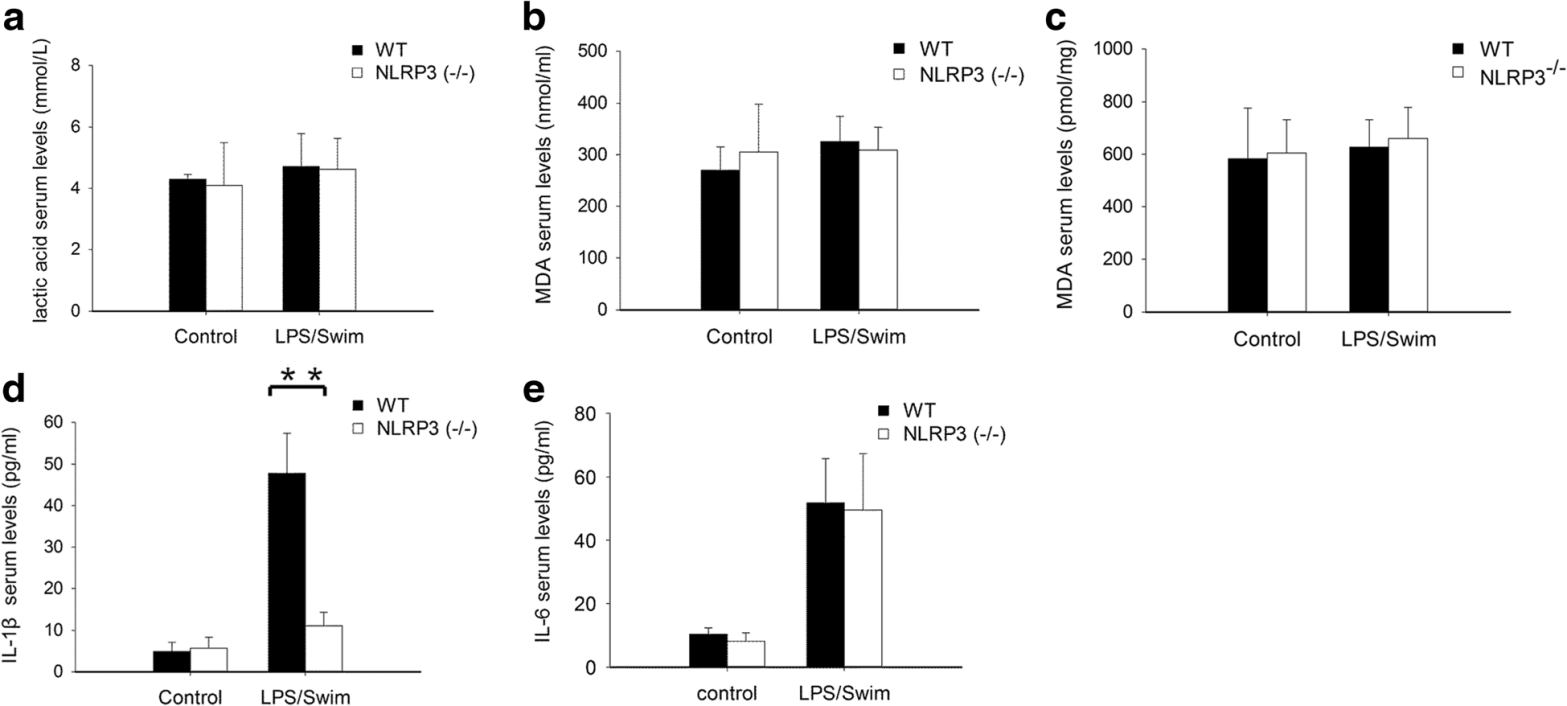
Effect of NLRP3 deficiency on biochemical parameters in the CFS model. There was no such effect on lactic acid (**a**) and MDA levels (**b** serum; **c** muscle). NLRP3 KO mice had attenuated IL-1β production (**d**) in the CFS model when compared with that of wild-type mice. IL-6 production was almost the same between NLRP3 KO mice and wild-type mice (**e**). The results are expressed as the mean ± SEM. **p < 0.01 (independent *t* test). (*n* = 8–10 mice per group)

We also examined the serum levels of lactic acid, MDA, IL-1β and IL-6 in the NLRP3 KO mice. There was no significant difference in the serum lactic acid (*p* = 0.87, Fig. 6a) or MDA levels in the NLRP3 KO mice compared with those of the wild-type mice (*p* = 0.49, Fig. 6b). We also did not detect significant differences in muscle tissue lactic acid or MDA levels between the NLRP3 KO mice and the wild-type mice (0.66 ± 0.04 pmol/mg vs. 0.63 ± 0.04 pmol/mg, Fig. 6c). By contrast, serum IL-1β levels were significantly decreased in the NLRP3 KO mice (11.12 ± 3.12 pg/ml) compared with those of the wild-type mice (47.83 ± 9.50 pg/ml, *p* < 0.01, Fig. 6d). IL-6 serum levels, on the other hand, were similar in NLRP3 KO mice (49.55 ± 17.77 pg/ml) and wild-type mice (57.53 ± 10.70 pg/ml, *p* = 0.33, Fig. 6e) after LPS/swim challenge.

### Effects of NLRP3 deficiency on caspase-1 activation and IL-1β production in LPS/swim stress-treated mice

**Fig. 7:**
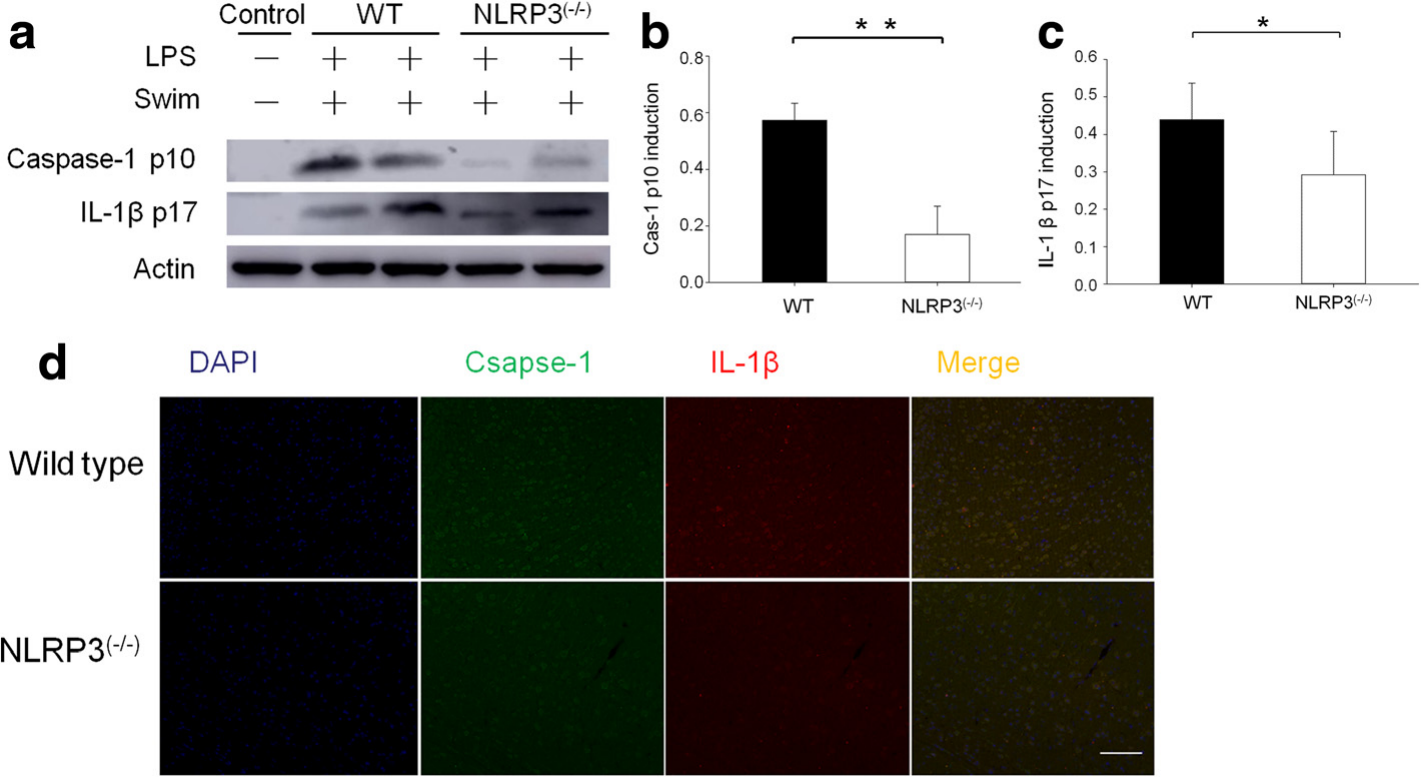
NLRP3 KO mice had reduced caspase-1 activation and IL-1β production in the diencephalon. The samples came from two mice per experiment, and the results are representative of three independent experiments (**a**). Cas-p10 induction (**b**) and IL-1β production (**c**) were quantified by measuring band intensities using ImageJ software. The values were normalized to β-actin. (**d**) DAPI (*blue*), Caspase-1 (*green*) and IL-1β (*red*) immunofluorescent staining in diencephalon sections; *scale bar*, 20 μm. The merged image shows the co-localisation of caspase-1 and IL-1β protein. **p < 0.01, *p < 0.05. Mean ± SEM of three samples

To investigate the mechanism underlying increased locomotor activity and increased fall-off time in the rota-rod test in NLRP3 KO mice, we further examined caspase-1 activation and IL-1β production in the mouse diencephalon following LPS/swim stress treatment. As shown in Fig. 7, the basal levels of caspase-1 activation and IL-1β production in the wild-type mouse diencephalon were almost undetectable (Fig. 7a). LPS/swim stress treatment induced strong caspase-1 activation and IL-1β production in the WT mice as described previously. By contrast, NLRP3 deficiency significantly decreased caspase-1 activation (*p* < 0.01, Fig. 7b) and IL-1β production (*p* < 0.05, Fig. 7c) under the same conditions. As shown in Fig. 7d, NLRP3 deletion resulted in decreased caspase-1 activation and IL-1β production following LPS/swim stress treatment. Co-localized expression of caspase-1 and IL-1β was also reduced in the NLRP3^(−/−)^ mice compared with that of the wild-type mice.

## Discussion

Experimental fatigue is classified into four categories: (1) physical fatigue, such as forced exercise and swimming; (2) mental fatigue; (3) environmental fatigue, such as heat exposure; and (4) immunologically induced fatigue [23]. Among these models, immunologically induced fatigue is usually considered to be associated with the neuroendocrine-immune interactions [24]. Lipopolysaccharide (LPS) treatment is commonly used to mimic bacterial infection and is also known to induce a behavioural sickness, which can be used as a model of fatigue [25, 26]. In this study, we established an LPS-induced fatigue model with the addition of swim stress, which is a combination of immunological and physical fatigue.

In the behavioural test, our study demonstrated that wild-type mice showed significantly decreased locomotor activity only following LPS/swim stress treatment. Mice treated with either LPS alone or subjected to only swim stress also exhibited decreased activity relative to controls, but this difference was not statistically significant (Fig. 1). These results conflict with those from previously published reports [27], and this discrepancy can possibly be explained by the low dose of LPS used here (3 mg/kg) and the relatively mild swim stress condition we applied. In our preliminary experiment, an intraperitoneal LPS injection at a dose of 10 mg/kg significantly affected locomotor activity without swim stress (data not shown). In addition, other published studies were mainly based on poly(I:C) intraperitoneal injection [27, 28]. Strain differences may also explain the different results, but apart from the knockout mouse strain, we did not use any other mouse strains besides C57BL/6 mice. These differences may also be attributed to various experimental designs. Our results indicated that although latent or mild infection itself was insufficient to induce fatigue, it could increase vulnerability to stress and exhaustion. Biochemical parameters including lactic acid, MDA, IL-1β and IL-6 are also involved in fatigue-associated illness [29]. So we measured their levels in our established mouse fatigue model. Lactic acid is a product of glycolysis under anaerobic conditions, and it accumulates during high-intensity physical exercise. As lactic acid accumulates, muscle tissue and blood pH decreases, which harms muscle tissue and causes fatigue [30, 31]. We tested serum lactic acid levels to reflect physical fatigue and found no significant difference between groups. Intense exercise may cause an imbalance between the body’s oxidation and anti-oxidation systems, thereby producing more ROS. ROS attack polyunsaturated fatty acids (PUFA), which can lead to lipid peroxidation [32]. MDA is one of the degradation products in the lipid peroxidation process [33]. Thus, MDA is an ideal parameter to measure and to understand oxidation status and the production of ROS. In light of CFS pathophysiology, we tested MDA levels in both serum and muscle tissue and found no significant difference in either tissue among the four groups. When compared with other groups, the LPS/swim stress mice produced more serum IL-1β and IL-6, which are two important inflammatory biomarkers (Fig. 2). Taken collectively, our results suggest that the fatigue symptom observed in the LPS/swim stress mice was mainly attributed to inflammation-induced fatigue rather than muscular weakness, muscular pain or lipid peroxidation.

The NLRP3 inflammasome has recently emerged as an unexpected sensor of metabolic danger and stress [34]. ROS production and mitochondrial dysfunction have been reported to trigger NLRP3 inflammasome activation [35]. Indeed, NLRP3 activation has been implicated in the development of many major diseases such as gout, type 2 diabetes, obesity-induced insulin resistance and depression [36, 37]. NLRP3 protein expression can be highly induced by stimulation with LPS through Toll-like receptor 4 (TLR-4), and IL-1β is the main cytokine produced by NLRP3 [38]. It has been reported that IL-1β levels in the brain or other tissues are highly correlated with CFS symptoms [39]. In this study, we investigated the role of NLRP3 inflammasome activation during LPS-induced fatigue. The NLRP3 inflammasome was strongly activated in LPS/swim stress mice, and exposure to swim stress significantly enhanced LPS-induced NLRP3 expression and activation (Fig. 4). Moreover, we found that NLRP3 activation was essential to fatigue pathogenesis, as shown by the attenuated behavioural performance observed in NLRP3 KO mice (Fig. 5). In Western blot and immunofluorescence analysis, the NLRP3 KO mice showed significantly decreased caspase-1 activation as well as reduced IL-1β production in the mouse diencephalon (Fig. 7). Interestingly, in the biochemical analysis, NLRP3 KO mice had significantly decreased the IL-1β serum levels, whereas the IL-6 levels in these mice were not different from those of the wild-type mice, which suggested that such fatigue model was mainly IL-1β-dependent. These results, together with other reports underlining the importance of IL-1β in CFS [10, 40], indicated that LPS-induced fatigue is an IL-1β-dependent disorder and that NLRP3/caspase-1 inhibition therapy may be a promising option for anti-fatigue therapy. To the best of our knowledge, this is the first study to report NLRP3 inflammasome activation in the CNS in a model of fatigue.

NLRP3 inflammasome activation is often described in terms of a two-step process requiring two signals [41]. For example, many initial signals, such as LPS, are used to prime the cell by inducing NLRP3 expression, and then a secondary signal forms the inflammasome complex and leads to the cleavage of caspase-1 and the maturation of IL-1β [16, 42]. In vitro, this signal distinction is often less clear [43, 44]. This is because the priming stimulus might itself lead to the release of activators such as ATP. In our results, although neither LPS treatment nor exposure to swim stress alone was able to trigger significant behavioural and biochemical changes, the combination of both could significantly promote NLRP3 activation in the mouse diencephalon. Our data are consistent with the two-step activation process. Our results showed slight caspase-1 p10 induction and IL-1β secretion in the LPS/no swim stress mice, and swim stress enhanced this effect similar to administering ATP in vitro. Overall, our study demonstrated that LPS/swim stress promotes robust NLRP3 inflammasome activation in the CNS.

## Conclusions

Here, we established an LPS-induced fatigue model in mice. We found decreased locomotor activity and motor performance in a rota-rod test of this model. The activation of the neural NLRP3/caspase-1 pathway is involved in the pathogenesis of this model. The results derived from the NLRP3 KO mice demonstrated that LPS-induced fatigue is an IL-1β-dependent process, which opens new avenues for NLRP3/caspase-1 inhibition therapy. Nevertheless, the need for more reliable animal models for fatigue study is urgent.

## Additional file

Additional file 1: Figure S1. The collected diencephalon region is indicated by a red circle. (TIF 5611.52 kb)

## Abbreviations

CDC: Centers for Disease Control and Prevention
CFS: chronic fatigue syndrome
CoQ10: coenzyme Q10
DAMPs: danger-associated molecular patterns
DAPI: (4′,6-diamidino-2-phenylindole)
IL-1β: interleukin-1β
IL-6: interleukin-6
LPS: lipopolysaccharide
MDA: malondialdehyde
NLRP3: NOD-like receptor family, pyrin domain containing 3
PBMC: peripheral blood mononuclear cells
PUFA: polyunsaturated fatty acids
ROS: reactive oxygen species
