# Supplementary figures and images for: Activation of the NLRP3 inflammasome in lipopolysaccharide-induced mouse fatigue and its relevance to chronic fatigue syndrome
Source: J Neuroinflammation. 2016 Apr 5;13:71. doi: 10.1186/s12974-016-0539-1 (PMC4822300; doi:10.1186/s12974-016-0539-1)

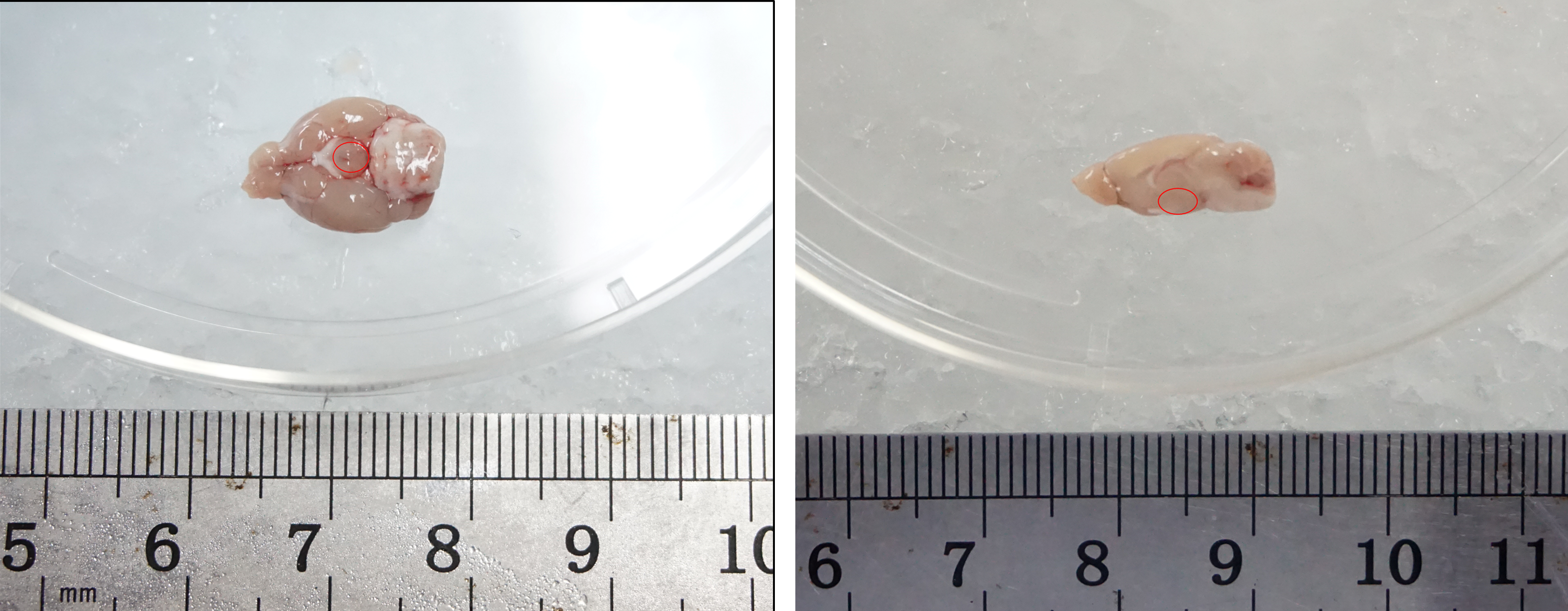

Supplement: Additional file 1: Figure S1. — The collected diencephalon region is indicated by a red circle. (TIF 5611.52 kb) [file 12974_2016_539_MOESM1_ESM.tif]
